# Association of Depression, Antidepressants With Atrial Fibrillation Risk: A Systemic Review and Meta-Analysis

**DOI:** 10.3389/fcvm.2022.897622

**Published:** 2022-05-11

**Authors:** Yonghui Fu, Shenghui Feng, Yingxiang Xu, Yuanjian Yang, Haibo Chen, Wenfeng He, Wengen Zhu, Kang Yin, Zhengbiao Xue, Bo Wei

**Affiliations:** ^1^Department of Psychiatry, Jiangxi Mental Hospital, Affiliated Mental Hospital of Nanchang University, Nanchang, China; ^2^Jiangxi Provincial Clinical Research Center on Mental Disorders, Nanchang, China; ^3^Department of Medical, Queen Mary School, Nanchang University, Nanchang, China; ^4^Jiangxi Key Laboratory of Molecular Medicine, The Second Affiliated Hospital of Nanchang University, Nanchang, China; ^5^Department of Cardiology, The First Affiliated Hospital of Sun Yat-sen University, Guangzhou, China; ^6^Department of Critial Care Medicine, The First Affiliated Hosptial of Gannan Medical University, Ganzhou, China

**Keywords:** atrial fibrillation, depression, antidepressants, risk factor, meta-analysis

## Abstract

**Background:**

Depression is a possible influence factor for the increased risk of incident atrial fibrillation (AF). Although several investigations have assessed their association, the results are still controversial. Therefore, we conducted a meta-analysis to evaluate the association between depression or using antidepressants and AF.

**Methods:**

We systemically performed the literature retrieval from two electronic databases PubMed and EMBASE until March 2022 to extract relevant data. The hazard ratios (HRs) and odds ratios (OR) from included studies with 95% confidence intervals (CIs) were adjusted into the risk ratio (RR) and pooled by using the random-effects model.

**Results:**

Totally 9 studies about the associations between depression or antidepressants and incident AF risk were included in this meta-analysis. Among them, 5 studies specifically analyzed the impact of antidepressants on the risk of AF. The outcomes of our analysis indicated that depression or depressive symptoms could increase AF risk (RR = 1.15, 95% CI, 1.03–1.27, *P* < 0.01). In addition, the use of antidepressants can also increase AF risk (RR = 1.16, 95% CI, 1.07–1.25, *P* < 0.001). These results remained unchanged when we remove the source of heterogeneity or adjust the analysis model into the fixed-effects model.

**Conclusions:**

Based on existing investigations, both depression and the use of antidepressants are closely related to the increase of incident AF risk.

## Introduction

Atrial fibrillation (AF) is the most prevalent cardiac arrhythmia with an age-related increase in incidence (1). It is strongly associated with stroke, heart failure morbidity (2–4), and increased mortality (5, 6). Early identification of high AF risk population is crucial for avoiding the adverse consequences related to AF. There are several factors have been identified to be related to the etiology of AF, including genetic factors, environmental factors, and other complications (7). However, more than one-third of the potential risk factor contributing to AF is still unexplained. Therefore, further investigations for additional AF risk factors including smoking, cardio-metabolic factors, and several psychological factors (8–10) need to be conducted.

In recent years, the association between depression and AF has been confirmed in several basic and epidemiological studies (11, 12). Theoretically, depression is closely related to the dysregulation of the hypothalamus-pituitary-adrenal (HPA) axis and inflammation. The hyperactivation of HPA axis could induce the consistent release of cortisol, which is also a marker of cortisol resistance (13). Cortisol resistance stimulates immune activation, then the expression level of some proinflammatory cytokines, including IL-2, IL-6, IL-12, and TNF-a will be increased. These cytokines act on the brain, developing some symptoms of depression in susceptible populations (14, 15), and are capable of producing systemic inflammation. The influenced HPA axis determines that depression is often accompanied by hypertension, metabolic syndrome, and obesity (16–18), which aggravate oxidative stress and inflammation in the body. Systemic inflammation increases the risk of AF by changing the electrophysiology (i.e., affecting calcium flowing), conduction and structural substrates of the atrial (19, 20). Moreover, depression may alter the sympathetic and parasympathetic balance to induce the decreased arrhythmic threshold (1), which also influences the atrial conductivity and structural integrity (14). Smoking as an accepted risk factor of AF, is more common in people with depression, since high negative affect and low positive affect in depression might raise the patients' dependence on nicotine (21). Nicotine has been reported with the function of promoting atrial structural remodeling and interstitial fibrosis (22). Therefore, depression is a potential factor for inducing new-onset AF.

As for antidepressants which are divided into three categories including selective serotonin reuptake inhibitor (SSRI), tricyclic antidepressants and monoamine oxidase inhibitor, their cardiotoxicity has also been reported in previous studies (23, 24). Tricyclic antidepressant mainly affects intraventricular conduction, which is characterized by prolonged PR, QRS and QT intervals on the electrocardiogram (ECG) (25). SSRI tends to increase serum serotonin, which then induces the elevation of intracellular calcium level (26, 27). As a result, the amplitude of the pacemaker is increased and potentially influences the heart rhythm (27). However, on the other hand, the use of antidepressants is capable of ameliorating the imbalance conditions of proinflammatory cytokines in depression (28–30), which may reduce the risk of depression-induced AF to a certain extent.

Considering that whether depression and the use of antidepressants could increase the risk of AF remains a controversial issue in previous studies, herein, we performed a meta-analysis including all of the existing studies to detect the association between AF risk and depression.

## Methods

This meta-analysis was based on the preferred reporting items for systematic review and meta-analysis (PRISMA) 2020 guidelines. Ethical approval was not provided since all data included in this study was from the published studies. The data, techniques, and materials that support the findings of this study will be available from the corresponding author according to reasonable requests.

### Literature Retrieval

The PubMed, and EMBASE electronic databases were selected for systemic search in this study. Two independent reviewers identified potentially eligible studies that reported the relationship between depression and the risk of AF. There were no language restrictions in the retrieval process. Terms used in screening include (atrial fibrillation OR atrial flutter) AND (depression OR depressive symptom OR antidepressant). The literature search strategy is resented in Supplementary Table 1, and the last retrieval was conducted in March 2022.

### Eligibility Criteria

Literatures meeting the following criteria were included in this study: (1) Studies that reported the relationship between depression, depressive symptoms or the use of antidepressants and the risk of incident AF; (2) Cohort or case-control studies included both comparison and control groups, and data were obtained through follow-up; (3) Studies defined the depression and depressive symptoms according to definite criteria. There was no limitation on the follow-up period. Specific literature forms including reviews, case reports, case series, editorials and meeting abstracts were excluded from this study. In addition, studies with insufficient clinical data were also decided for exclusion.

### Study Selection and Data Extraction

Two authors extracted data independently through screening the literature titles and abstracts. Then the full-text screening was conducted to detect whether the literature met the inclusion criteria. All discrepancies were resolved by discussing with the third researcher. If multiple studies from the same data source were suitable for this meta-analysis, only the study that best matched the eligibility criteria were included. Studies with later publication years and longer follow-up periods were preferentially included.

The relevant information of each study was recorded, including the first author, publication year, data source, information of participants (sample size, age, and sex), the definition of depression, adjusted confounders, and follow-up period. For the included studies that reported the adjusted RRs by using multiple models, only the most adjusted data was used in this meta-analysis.

### Study Quality Assessment

The quality of eligible studies was assessed by using the Newcastle-Ottawa Scale tool, which covers three aspects, ranging from 0 to 9 stars: the cohort selection (0–4 stars), cohort comparability (0–2 stars), and the evaluation of study outcomes (0–3 stars). Studies with NOS results of <6 points were considered as low quality.

### Statistical Analysis

The statistical analyses were performed by using the Review Manager version 5.4 software (The Cochrane Collaboration 2020, Nordic Cochrane Centre Copenhagen, Denmark; https://community.cochrane.org/). The statistical heterogeneity was evaluated by *I*^2^ statistic and the Cochrane Q-test. Either *P* < 0.1 for the Q-test or *I*^2^ ≥ 50% was considered as the indication of substantial heterogeneity. For the results, *P* < 0.05 was considered to have statistical significance. To the extent possible, we used the same treatment effect indicators in cohort and case-control studies. Reported maximally adjusted hazard ratios (HRs) or odds ratios (ORs), and 95% confidence intervals (CIs) were extracted. For studies that reported multiple categories of psychological factors (e.g., the degree of depressive symptoms), HR in the most severe category was used. In the results section, we refer to all relative effects metrics as “risk ratios” (RRs), which do not affect the study results or their interpretation. The corresponding natural logarithm (Ln[RR]) and standard error (SE) of each investigation were used for calculation. Given the intrinsic heterogeneity of these included studies, the inverse-variance weighted random-effects model was applied to pool the Ln [RR] and its SE. The publication bias was assessed by funnel plots as well as Egger's and Begg's tests.

## Results

### Study Selection

**Figure 1:**
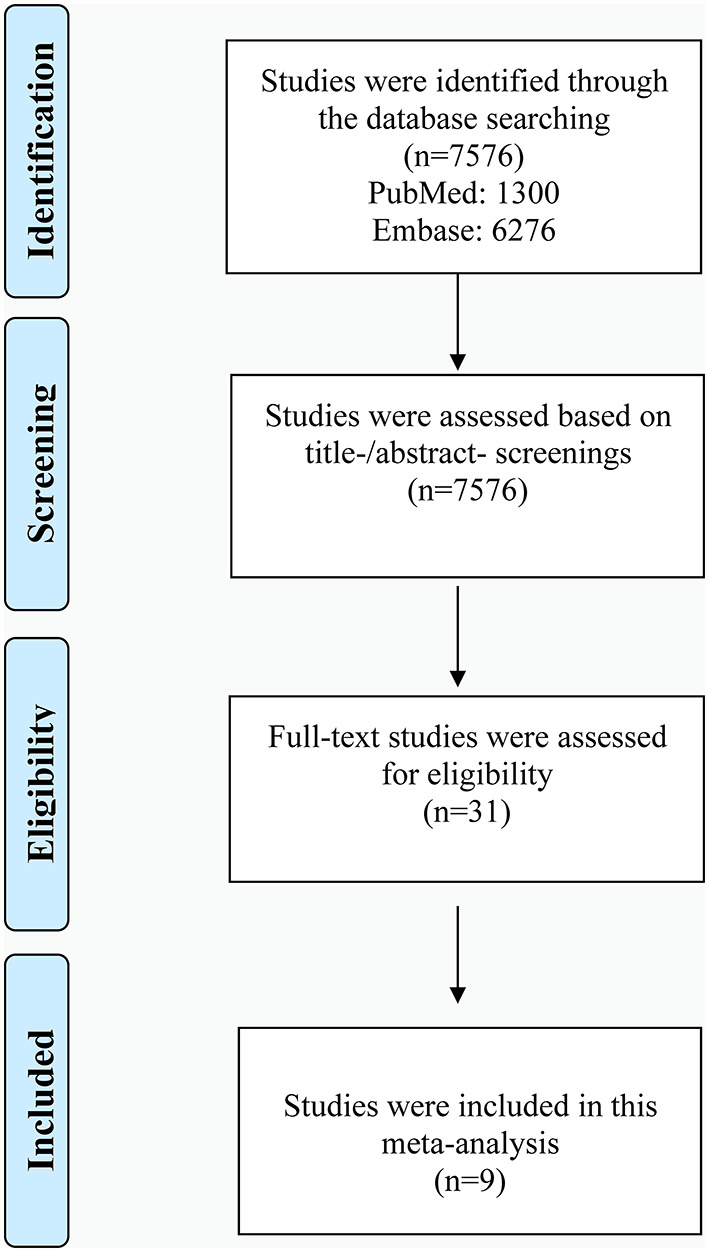
Diagram of study selection process of our meta-analysis.

Shown in Figure 1 is the literature retrieval process. Totally, 7,576 studies were obtained from initial online searching. Among them, 1,300 studies were from PubMed and 6,276 studies from Embase database. All of these studies were assessed based on title/abstract screening. Then 31 studies underwent full-text review for eligibility assessment. Based on predefined criteria, finally, 9 eligible studies were included in our meta-analysis (31–39). The diagnostic basis of depression and AF were clearly defined in all included articles. Exhibited in Table 1 is the baseline information of participants, while the diagnostic methods for AF in included studies are presented in Supplementary Table 2. As shown in Supplementary Table 3, all of the included studies in our investigation were considered as moderate to high quality.

**Table 1:** Baseline patient characteristics of included studies.

**Included studies**	**Data source**	**Study type**	**Research object**	**Sample size (*n*)**	**Mean age (*y*)**	**Sex**	**AF cases**	**Definition of depression**	**Adjusted for confounders**	**Follow-up (*y*)**
Whang et al. (39)	The Women's Health Study; United States	Cohort; 2004.03–2010.03	Female health professionals	30,746	59.0	Females	771	MHI-5 score <53, antidepressant use, or both	Age, race, BMI, hypertension, DM, hypercholesterolemia, smoking, alcohol intake, kilocalories from exercise, randomized treatment assignment	10.4
Egeberg et al. (32)	Danish National Patient Register; Denmark	Cohort; 1997.01–2011.12	Patients with psoriasis	67,853	42.9	Both	403	Antidepressant use	Age, sex, socioeconomic status, comorbidities, concomitant medication	5.1
Lapi et al. (38)	UK Clinical Practice Research Datalink	Nested case-control; 1993.01–2010.12	New users of antidepressants previously diagnosed with depression and/or anxiety	116,125	43.5	Both	1,270	Antidepressant use	Age, sex, cigarette smoking, BMI, alcohol use, indication of antidepressant use, comorbidities, concomitant medication	5.8
Fenger-Grøn et al. (34)	Nationwide register; Denmark	Cohort; 2000–2013	All Danes initiating antidepressant treatment	785,254	NA	Both	NA	Antidepressant use	Age, sex, DM, marital status, ischaemic heart disease, dyslipidaemia, hypertension, HF, stroke, peripheral artery disease, anemia, thyroid disorder, chronic kidney disease, schizophrenia or schizoaffective disorder, bipolar affective disorder dementia, alcohol abuse and/or other substance abuse	0.5–1.0
Garg et al. (36)	Multi-Ethnic Study of Atherosclerosis; United States	Cohort; 2000–2002	General population	6,644	62.0	Both	875	CES-D score, antidepressant use, or both	Age, sex, race, education, income, clinic site, cigarette smoking, BMI, height, DM, glucose, SBP, moderate and vigorous physical activity, statin use, antihypertensive use, current alcohol use	12.9
Feng et al. (33)	The third Nord-Trøndelag Health (HUNT 3) study; Norway	Cohort; 2006.10–2008.06	General population	37,402	53.4	Both	1,433	HADS-D ≥ 11	Age, sex, weight, height, smoking status, occupation, marital status, physical activity, alcohol consumption, chronic disorders, metabolic components (i.e., blood glucose, blood pressure, triglycerides, high-density lipoproteins and C-reactive protein)	8.1
Ditmars et al. (31)	The longitudinal Vietnam Era Twin Study of Aging (VETSA)	Cohort; 1965–1975	US military	787	41.4	Males	NA	DIS-III-R	NA	27.0
Garg et al. (35)	The Atherosclerosis Risk in Communities (ARIC) study; United States	Cohort; 1990–1992	General population	11,445	58.8	Both	2220	Antidepressant use	Age, sex, race-center, education, height, weight, cigarette smoking, DM, SBP, DBP, anti-hypertensive medication, total cholesterol, high-density lipoprotein, physical activity, alcohol consumption, coronary heart disease, HF, left ventricular hypertrophy, stroke	23.4
Kim et al. (37)	Korean National Health Insurance Service (K-NHIS) database	Cohort; 2002.01–2008.12	Nationwide health checkup in 2009	5,031,222	47.0	Both	78,262	ICD-10 codes	Age, sex, BMI, smoking status, alcohol consumption status, regular physical activity, income level, DM, hypertension, dyslipidemia, HF, thyroid disease, depression as a time-varying covariate	10.0

*MHI-5, 5-item Mental Health Inventory; CES-D, Center for Epidemiologic Studies Depression Scale; HADS, Hospital Anxiety and Depression Scale; DIS-III-R, the National Institute of Mental Health Diagnostic Interview Schedule, Version III, Revised; DM, diabetes mellitus; SBP, systolic blood pressure; DBP, diastolic blood pressure; BMI, body mass index; HF, heart failure; NA, not available*.

### Relationship Between Depression and AF

**Figure 2:**
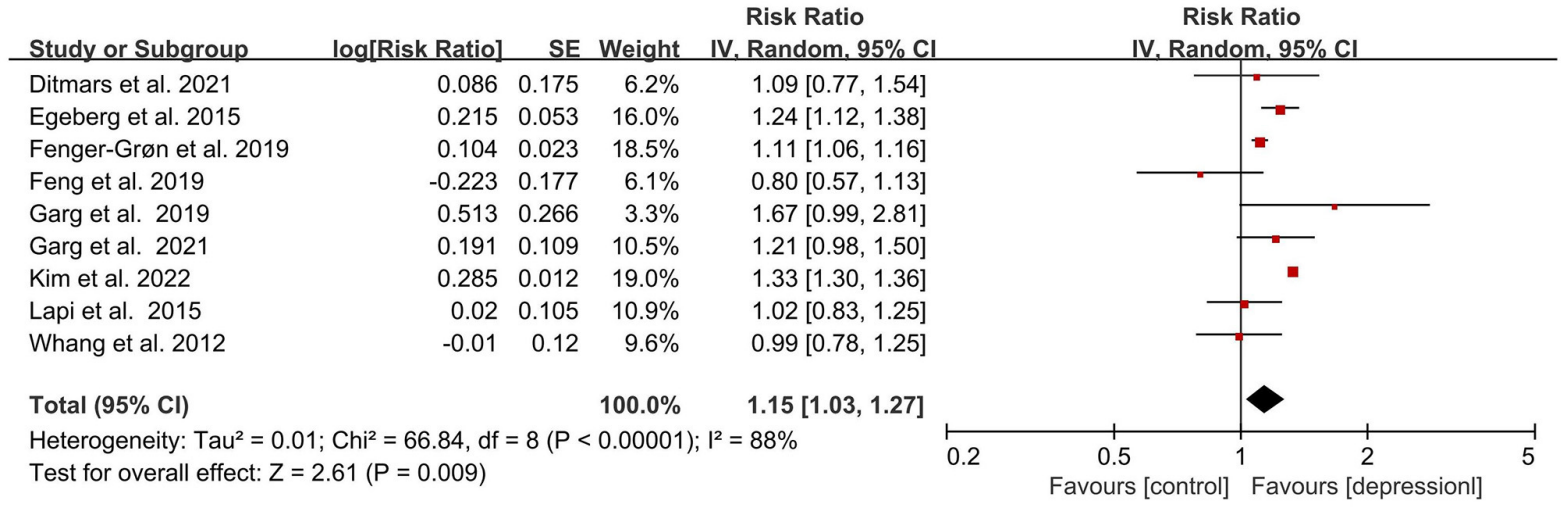
Forest plot for association of depression with atrial fibrillation risk. SE, standard error; CI, confidence interval; IV, inverse of the variance.

All of our eligible studies examined the association between depression and the risk of incident AF. Among them, most of the studies indicated that depression was related to the increase of AF risk. Only studies from Whang et al. and Feng et al. reported that there was no evidence of an association between the increased AF risk and depression (33, 39). Presented in Figure 2 is the outcome of our meta-analysis, which indicates that depression or depressive symptoms could increase AF risk (RR = 1.15, 95% CI, 1.03–1.27, *P* < 0.01). However, this result represented relatively high heterogenicity (*I*^2^ = 88%). In order to detect the source of heterogeneity, we screened and analyzed all of the data in the included studies by the exclusive method. As shown in Supplementary Figure 1, after removing the data from Kim et al., the heterogeneity is acceptable and the final outcome was not influenced (RR = 1.12, 95%CI, *I*^2^ = 42, *P* < 0.01). Also, this result was not changed when we adjusted the analysis into the fixed-effects model (RR = 1.27, 95%CI, 1.24–1.30, *P* < 0.001; Supplementary Figure 2).

### Relationship Between Antidepressants and AF

**Figure 3:**
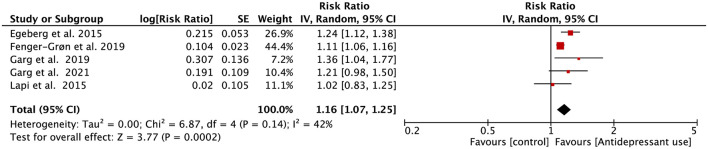
Forest plot for association of antidepressant use with atrial fibrillation risk. SE, standard error; CI, confidence interval; IV, inverse of the variance.

Five eligible studies reported the associations between the use of antidepressants and AF risk. Four of them reported that the risk of incident AF in antidepressant users was substantially increased. Only the study from Lapi et al. indicated that exposure to antidepressants is not associated with the increased risk of AF (38). As shown in Figure 3, the outcome of our meta-analysis support that the risk of incident AF was significantly increased in the antidepressant using population (RR = 1.16, 95% CI, 1.07–1.25, *P* < 0.001). The heterogenicity of included studies was acceptable (*I*^2^ = 42%). This result remained unchanged when we adjusted the analysis model into the fixed-effects model (RR = 1.13, 95%CI, 1.09–1.18, *P* < 0.001; Supplementary Figure 3).

## Publication Bias

As for bias risk assessment, the corresponding funnel plots for depression and antidepressant related studies were included in Supplementary Figures 4, 5. We also adopted Egger's and Begg's test to detect the presence of publication biases, which were presented in Supplementary Figures 6, 7. The results showed that the *P*-values of the two groups were ≥ 0.1, indicating that the bias risk of included studies in our meta-analysis was relatively lower.

## Discussion

Based on previous studies, we conducted this meta-analysis to evaluate the association between depression, antidepressants and the risk of AF. After pooling the data from 9 included observational studies, the primary outcomes of our investigation indicate that both depression and the use of antidepressants are capable of increasing the risk of incident AF.

Previous studies suggested that the occurrence of depression or depressive symptoms is closely related to some immune signaling, especially proinflammatory cytokines IL-2, IL-6, IL-12 and TNF-a (14, 40, 41). These cytokines are capable of inducing systemic inflammation, which is a potential risk factor of AF since the inflammatory cell infiltration has been observed in the atrial of AF patients (42, 43). According to the studies investigating the relationship between depression and AF, these two symptoms are in a comorbid state (44, 45). However, earlier meta-analysis pointed out that depression is related to the increased risks of sudden cardiac death, ventricular tachycardia/ventricular fibrillation, and AF recurrence (19), but the association between depression and incident AF was not considered to exist (10, 19). After reanalysis, we considered that some non-negligible limitations might affect their accuracy. As for the study from Shi et al., only two studies specific for depression and AF were included. In the study from Whang et al., the selection of the included population, such as only women or mainly white people may lead to the deviation of outcomes. In addition, people identified as depression through questionnaires were also included in this meta-analysis, which may induce a bias in the diagnosis of depression. Fu et al. only included 5 studies in their study, the majority of the study participants were from the US or Europe, the ethnic interference of study outcome also cannot be fully eliminated.

In addition, whether the use of antidepressants could impact AF risk was also a controversial issue in the previous studies (38, 46). Theoretically, tricyclic antidepressants affect cardiac conduction and cardiotoxicity (25). As for the use of SSRI antidepressants, patients' serum serotonin levels are elevated during medication. Serotonin promotes calcium overload, which may trigger focal atrial extrasystoles and increase the risk of AF (27, 47). Both of these two types of drugs are associated with prolonged QTc and increased risk of arrhythmias (48), but studies focusing on the risk of incident AF increased by monoamine oxidase inhibitors are relatively fewer. However, studies have suggested that treating with antidepressants may alter the imbalance conditions of inflammatory cytokines in depression (28–30). Our findings provide strong evidence for the view that antidepressants increase the risk of incident AF, which indicates that the prevention of AF in patients with depression deserves further research in the future.

After incorporating all of the latest relevant studies, the outcome of our analysis confirms the theoretical link between depression and incident AF. However, the substantial heterogeneity of our investigation still exists. After removing the study from Kim et al. (37), the outcome was not influenced and the heterogeneity is acceptable. The sources of heterogeneity are speculated as follows: (1) The race difference between this study and other included studies was non-negligible. Kim et al. obtained data from the Korean National Health Insurance Service database, whose study population was Asian. Except for this study, the investigation participants of other included studies in our meta-analysis were from the United States or Europe. The risk of incident AF has shown differences among different ethnic groups (49, 50). (2) This study was based on insurance claim data of ICD-10 codes for depression and AF, rather than incident AF diagnosis during follow-up. The different outcome definitions between this study and the rest studies may also lead to the existence of heterogeneity.

Previous investigations have pointed out the molecular mechanisms by which depression and the use of antidepressants might increase the risk of incident AF. These mechanisms laid a theoretical foundation for our research. After pooling all of the data from existing investigations, the results of our study quantitatively confirm that patients with depression and antidepressant users have an increased risk of new-onset AF by 15 and 16%, respectively. These data suggest that the cardiovascular health of patients with depression deserves special attention, and it is necessary to strengthen the cooperation between cardiologists and psychiatrists in the process of depression treatment. For future study, the effects of different types of antidepressants on incident AF deserves further exploration, which is helpful to formulate a more reasonable management plan for patients with depression.

## Limitations

Although our study has included as much data as possible and tried to avoid the influence of confounding factors, several potential limitations still exist. First of all, the substantial heterogeneity is relatively high in our study. This may be induced by analysis strategies and participant features. However, due to the limited data, subgroup analysis based on these factors cannot be carried out. Secondly, the evaluation criteria for depressive symptoms and incident AF were inconsistent, which might induce the existence of small deviations in the diagnosis of depression. Thirdly, most of the data were obtained from observational cohort studies. Although most of them were adjusted for multivariable confounding factors, the corresponding information of each included literature was not completely consistent, and the residual confounding factors cannot be completely excluded. Future studies can use the method of propensity score matching to make the baseline data of participants more comparable. Finally, the number of existing studies in the antidepressant group was relatively limited, which does not support the subgroup analysis of different types of antidepressants. Future studies can assess the effects of different types of antidepressants on incident AF after incorporating more eligible data.

## Conclusion

Based on existing investigations, both depression and the use of antidepressants are related to the increased risk of incident AF. Further study is needed to conduct more subgroup analysis and confirm our findings.

## Data Availability Statement

The original contributions presented in the study are included in the article/Supplementary Material, further inquiries can be directed to the corresponding author/s.

## Author Contributions

All authors listed have made a substantial, direct, and intellectual contribution to the work and approved it for publication.

## Funding

This study was supported by the National Natural Science Foundation of China [No. 31960146] and Special fund for postgraduate innovation in Jiangxi Province (YC2012-B011).

## Conflict of Interest

The authors declare that the research was conducted in the absence of any commercial or financial relationships that could be construed as a potential conflict of interest.

## Publisher's Note

All claims expressed in this article are solely those of the authors and do not necessarily represent those of their affiliated organizations, or those of the publisher, the editors and the reviewers. Any product that may be evaluated in this article, or claim that may be made by its manufacturer, is not guaranteed or endorsed by the publisher.
