# Supplementary material for: Association of Depression, Antidepressants With Atrial Fibrillation Risk: A Systemic Review and Meta-Analysis
Source: Front Cardiovasc Med. 2022 May 11;9:897622. doi: 10.3389/fcvm.2022.897622 (PMC9130653; doi:10.3389/fcvm.2022.897622)
Supplement: Supplementary file 1 [file Data_Sheet_1.docx]

**Supplemental Table 1. The search strategies up to March, 06 2022**

| **PubMed** | **Terms** | **No.** |
| --- | --- | --- |
| #1 | atrial fibrillation | 96,529 |
| #2 | atrial flutter | 10,176 |
| #3 | #1 or #2 | 100,101 |
| #4 | depression | 567,766 |
| #5 | depressive symptom | 456,105 |
| #6 | antidepressant | 188,622 |
| #7 | #4 or #5 or #6 | 686,624 |
| #8 | #3 and #7 | 1,300 |
| **Embase** |  |  |
| #1 | atrial fibrillation | 202,219 |
| #2 | atrial flutter | 10,608 |
| #3 | #1 or #2 | 206,303 |
| #4 | depression | 777,377 |
| #5 | depressive symptom | 4,315 |
| #6 | antidepressant | 547,619 |
| #7 | #4 or #5 or #6 | 1,185,693 |
| #8 | #3 and #6 | 6,276 |

**Supplemental Table 2. Evaluation of AF incidence**

| **Study (Year)** | **Assessment criteria** |
| --- | --- |
| Ditmars (2021) | self-reported doctor diagnosis |
| Egeberg et al. 2015 | ICD-8 codes 427.94 and 427.95, and ICD-10 code I48 |
| Feng (2019) | ICD-10 code I48 |
| Fenger-Grøn (2019) | ICD-8 code 427.93 or 427.94 or ICD-10 code I48 |
| Garg (2021) | ICD-9-CM code 427.31 or 427.32 |
| Garg (2019) | ECGs, ICD-9-CM code 427.31 or 427.32, Claim data |
| Kim (2022) | insurance claim with ICD-10 codes for depression |
| Lapi (2015) | Read codes used in CPRD studies coupled with specific cardiac surgical or monitoring procedures and interventions (ie, AF monitoring, ECG confirming a diagnosis of AF or flutter) |
| Whang (2012) | Medical records |

**ICD: International classification of diseases; ECG: electrocardiogram; CPRD: clinical practice research datalink**

**Supplemental Table 3. NOS score**

|  | Selection | | | | Comparability | Outcome | | |  |
| --- | --- | --- | --- | --- | --- | --- | --- | --- | --- |
| Cohort Study | Representativeness of the exposed cohorts | Selection of the non-exposed cohort | Ascertainment of exposure | Demonstration that outcome of interest was not present at start of study | Comparability of cohorts on the basis of the design or analysis | Assessment of outcome | Was follow-up long enough for outcomes to occur | Adequacy of follow up of cohorts | Quality score |
| Ditmars (2021) |  | ★ | ★ | ★ | ★★ |  | ★ |  | 6 |
| Egeberg（2015） | ★ | ★ | ★ | ★ | ★★ | ★ | ★ |  | 8 |
| Feng (2019) | ★ | ★ | ★ | ★ | ★★ | ★ | ★ |  | 8 |
| Fenger-Grøn (2019) | ★ | ★ | ★ | ★ | ★ | ★ | ★ | ★ | 8 |
| Garg (2021) | ★ | ★ | ★ | ★ | ★★ | ★ | ★ | ★ | 9 |
| Garg (2019) | ★ | ★ | ★ | ★ | ★★ | ★ | ★ | ★ | 9 |
| Kim (2022) | ★ | ★ | ★ | ★ | ★ | ★ | ★ |  | 7 |
| Whang (2012) |  | ★ | ★ | ★ | ★★ | ★ | ★ |  | 7 |
| Case-control Study | Is the case definition adequate | Representativeness of the cases | Selection of Controls | Definition of Controls | Comparability of cases and controls on the basis of the design or analysis | Ascertainment of exposure | Same method of ascertainment for cases and controls | Non-Response rate | Quality score |
| Lapi (2015) | ★ | ★ | ★ | ★ | ★★ | ★ | ★ |  | 8 |


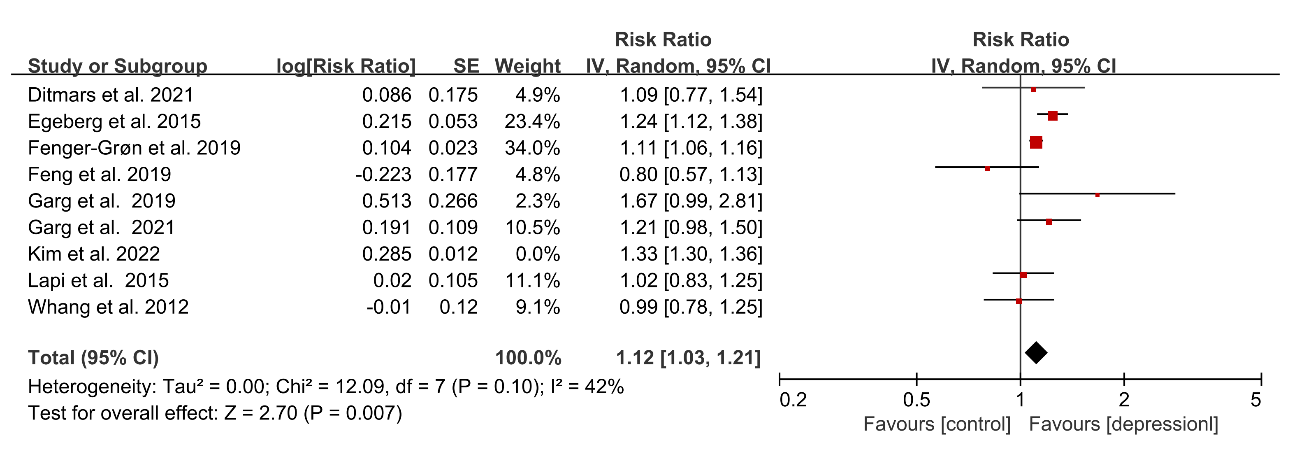


**Supplemental Figure 1. Forest plot for association of depression with atrial fibrillation risk (excluding the study of Kim et al)**

SE=standard error; CI=confidence interval; IV=inverse of the variance


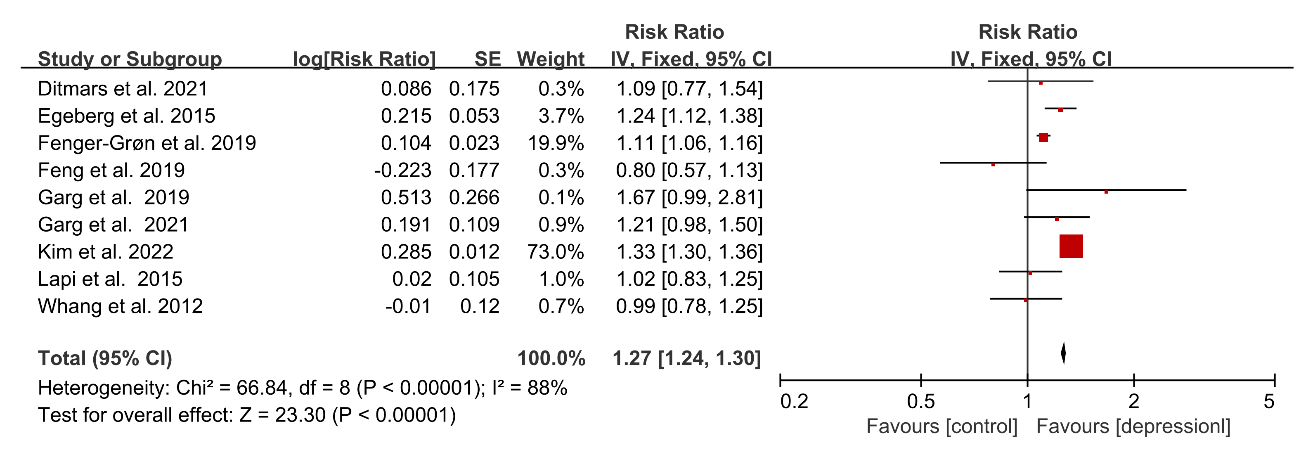


**Supplemental Figure 2. Forest plot for association of depression with atrial fibrillation risk (fixed model)**

SE=standard error; CI=confidence interval; IV=inverse of the variance


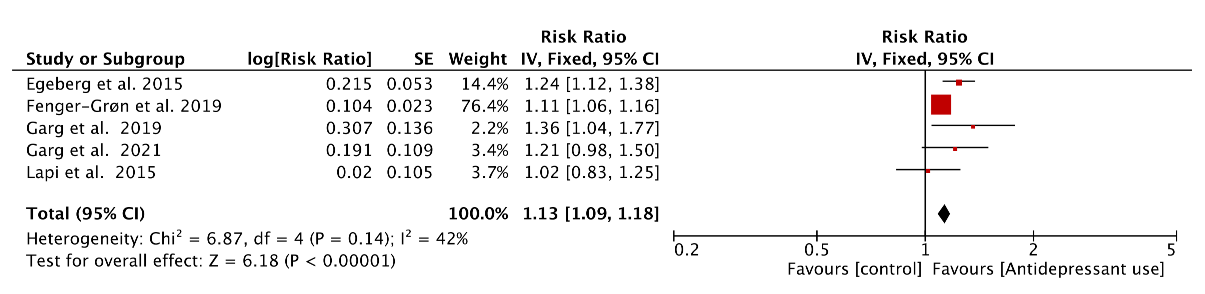


**Supplemental Figure 3. Forest plot for association of antidepressant use with atrial fibrillation risk (fixed model)**

SE=standard error; CI=confidence interval; IV=inverse of the variance


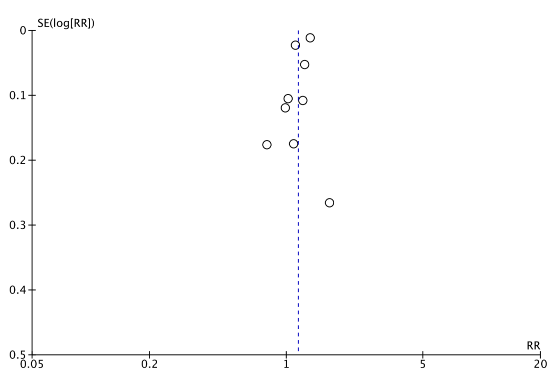


**Supplemental Figure 4. Funnel plot for association of depression with atrial fibrillation risk**


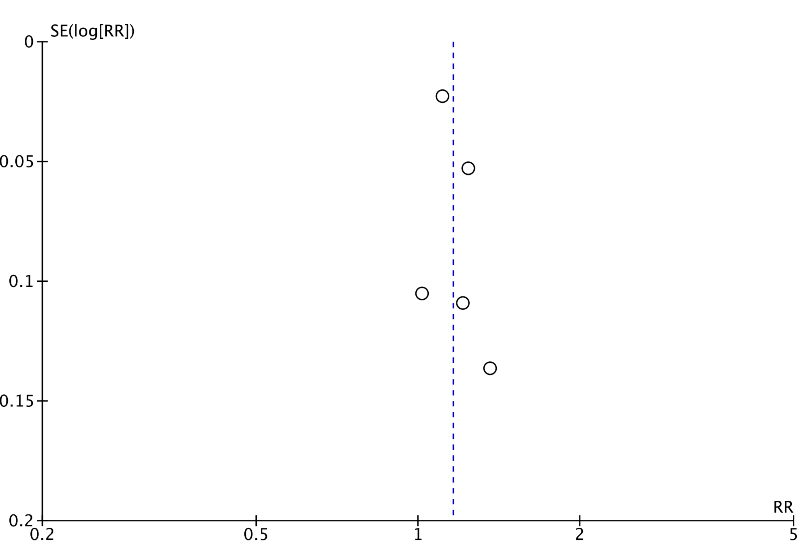


**Supplemental Figure 5. Funnel plot for association of antidepressant use with atrial fibrillation risk**


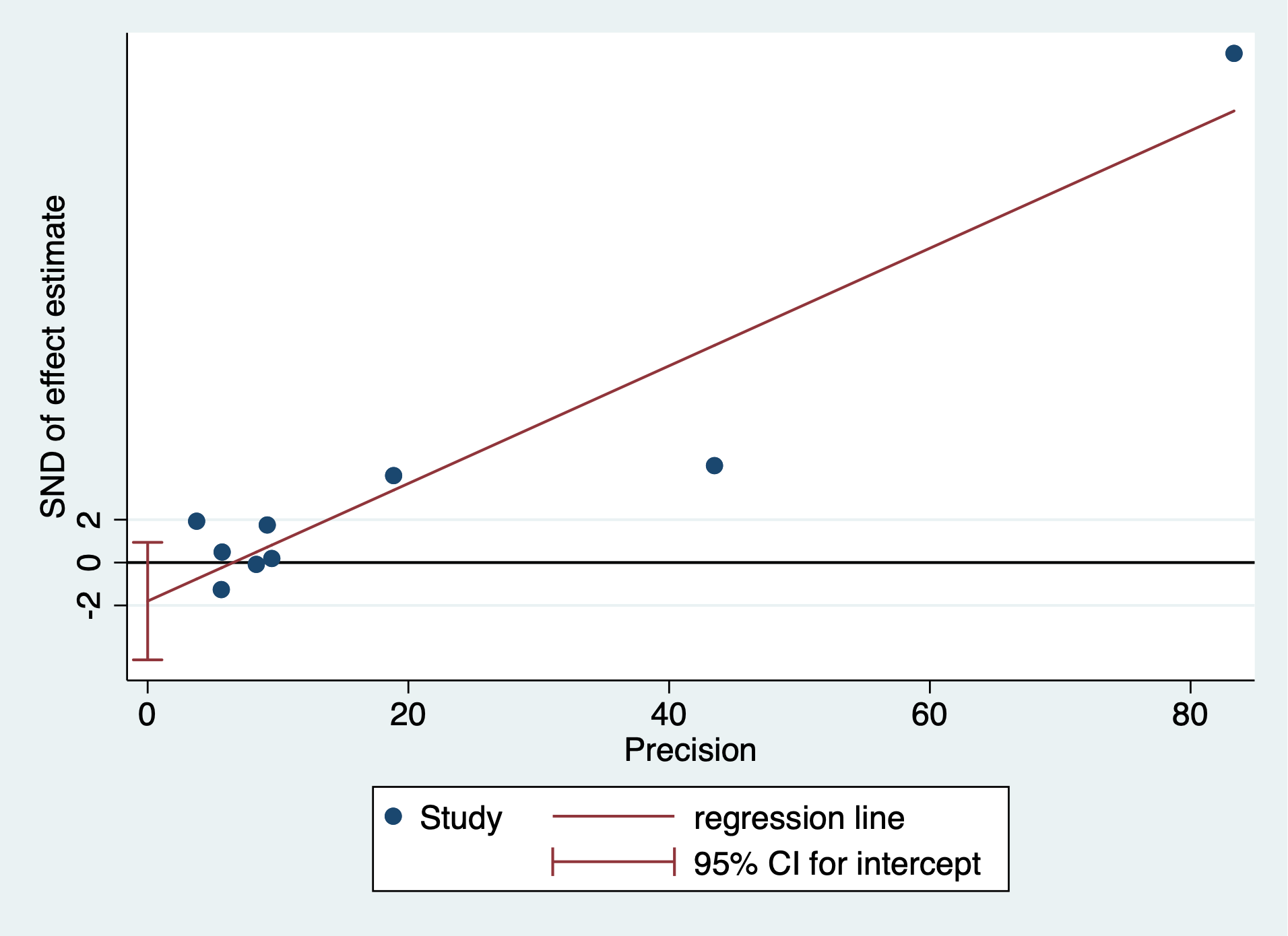


**Supplemental Figure 6. Egger test (P=0.641) for association of depression with atrial fibrillation risk**

**Note: Begg test (P=1.0)**


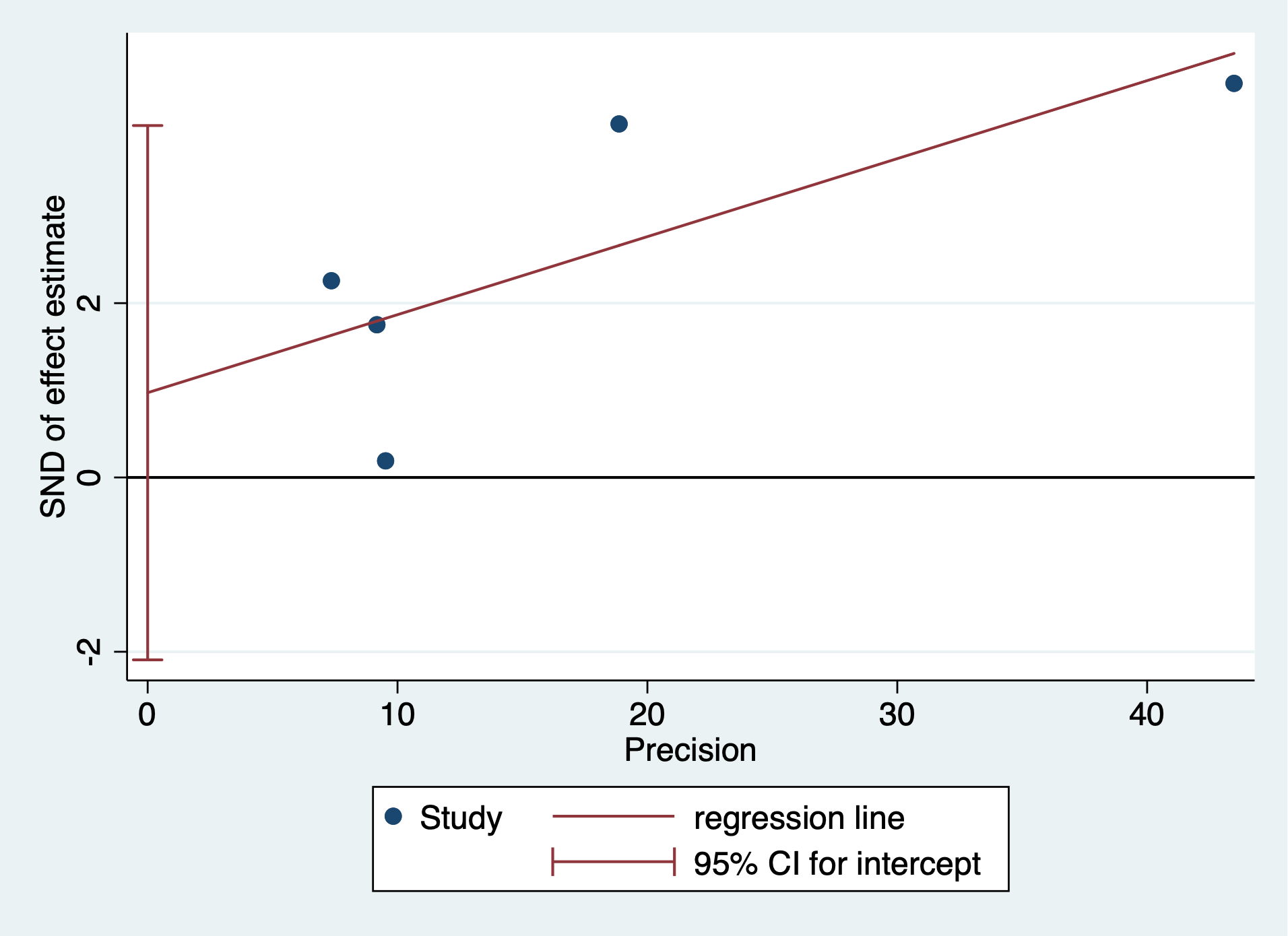


**Supplemental Figure 7. Egger test (P=0.387) for association of antidepressant use with atrial fibrillation risk**

**Note: Begg test (P=0.462)**
